# RNA-Seq differential expression analysis: An extended review and a software tool

**DOI:** 10.1371/journal.pone.0190152

**Published:** 2017-12-21

**Authors:** Juliana Costa-Silva, Douglas Domingues, Fabricio Martins Lopes

**Affiliations:** 1 Department of Computer Science, Bioinformatics Graduate Program, Federal University of Technology - Paraná, Cornélio Procópio, PR, Brazil; 2 Department of Botany, Institute of Biosciences, São Paulo State University, UNESP, Rio Claro - SP, Brazil; New Jersey Institute of Technology, UNITED STATES

## Abstract

The correct identification of differentially expressed genes (DEGs) between specific conditions is a key in the understanding phenotypic variation. High-throughput transcriptome sequencing (RNA-Seq) has become the main option for these studies. Thus, the number of methods and softwares for differential expression analysis from RNA-Seq data also increased rapidly. However, there is no consensus about the most appropriate pipeline or protocol for identifying differentially expressed genes from RNA-Seq data. This work presents an extended review on the topic that includes the evaluation of six methods of mapping reads, including pseudo-alignment and quasi-mapping and nine methods of differential expression analysis from RNA-Seq data. The adopted methods were evaluated based on real RNA-Seq data, using qRT-PCR data as reference (gold-standard). As part of the results, we developed a software that performs all the analysis presented in this work, which is freely available at https://github.com/costasilvati/consexpression. The results indicated that mapping methods have minimal impact on the final DEGs analysis, considering that adopted data have an annotated reference genome. Regarding the adopted experimental model, the DEGs identification methods that have more consistent results were the limma+voom, NOIseq and DESeq2. Additionally, the consensus among five DEGs identification methods guarantees a list of DEGs with great accuracy, indicating that the combination of different methods can produce more suitable results. The consensus option is also included for use in the available software.

## Introduction

High-throughput sequencing has become the main choice to measure expression levels, i.e., RNA-Seq [1]. RNA-Seq can be performed without prior knowledge of the reference or sequence of interest and allows a wide variety of applications such as: ‘de novo’ reconstruction of the transcriptome (without a reference genome), evaluation of nucleotide variations, evaluation of methylation patterns [2], to cite a few.

RNA-seq technology has some advantages over the cDNA microarrays, such as the high level of data reproducibility through lanes and flow-cells, which reduces the number of technical replicates for the experiments. Besides, RNA-seq allows to identify and quantify the expression of isoforms and unknown transcripts [3]. Regarding the increasing popularity of high-throughput sequencing methodologies, the cost of next-generation sequencing experiments has dropped considerably. However, a clear understanding about the qualitative and quantitative analysis of RNA-Seq has not yet been achieved, especially when compared to older methodologies such as cDNA microarray [4].

In general, the RNA-Seq technology is very useful for differential expression analysis involving some specific conditions [5], in which is commonly adopted five steps [6, 7]. First, the RNA samples are fragmented into small complementary DNA sequences (cDNA) and then sequenced from a high throughput platform. Second, the small generated sequences are mapped to a genome or transcriptome. Third, the expression levels for each gene or isoform are estimated. Fourth, the mapped data are normalized and, e.g. using statistical and machine learning methods, the differentially expressed genes (DEGs) are identified. Finally, the relevance of the produced data is finally evaluated from a biological context [8]. With the increasing popularity of RNA-Seq technology, many softwares and pipelines were developed for differential gene expression analysis from these data.

The methods for differential gene expression analysis from RNA-Seq can be grouped into two main subsets: parametric and non-parametric. Parametric methods capture all information about the data within the parameters. In these cases, it is possible to predict the value of unknown data from observing the adopted model and its parameters. When parametric methods are applied to differential gene expression assume that, usually after a normalization, each expression value for a given gene is mapped into a particular distribution, such as Poisson [9–11] or negative binomial [12–14]. On the other hand, non-parametric methods can capture more details about the data distribution, i.e., not imposing a rigid model to be fitted. It is possible because non-parametric models take into consideration that data distribution cannot be defined from a finite set of parameters, thus the amount of information about the data can increase with its volume.

Regarding the RNA-Seq differential expression analysis, some tools such as edgeR [13] and baySeq [11], adopt the negative binomial model as the main approach. Other software tools, such as NOIseq [15] and SAMseq [16], adopt non-parametric methods. Some methods are based on transcript detection, which have been developed in order to identify unknown transcripts or isoforms and also can be applied to the identification of DEGs, such as EBSeq [17] and Cuffdiff2 [18]. Nowadays, there is not a consensus about which methodology is most appropriate or which approach ensures the validity of the results in terms of robustness, accuracy and reproducibility. This topic in Bioinformatics research is still developing [5, 19, 20].

Some research effort were developed in order to evaluate the statistical methods of normalization and detection of DEGs and the influence of the libraries preparation on the results [10], to evaluate the methodologies of differential expression analysis by considering microorganism, including the mapping methods used for the analysis [21] and to evaluate the softwares and pipelines with simulated data [20, 22].

In particular, Rapaport *et al.* [23] evaluated a compendium of softwares for differential expression analysis in real data sets, considering the characteristics of the analysis such as accuracy, normalization, detection of DEGs and conditions without detected expression. Zhang *et al.* [5] evaluated the influence of the number of replicates, sequencing coverage and comparing groups. Guo *et al.* indicated that the ranking between three methods of DEGs identification can generate a more accurate identification [24]. Li *et al.* [8] evaluated standardization methods for DEG detection, indicating that the joining of two standardization methods led to better results. Seyednasrollah *et al.* [25] presented a comparison of eight software tools for DEG analysis in real data. Germain *et al.* [26] presented a work regarding the steps of RNA-Seq data analysis, comparing different methods of transcripts mapping and quantification, also presenting an on-line tool for the adopted methods comparison.

Recently, Yu *et al.* [27] presented a procedure based on simulation by adopting a negative binomial distribution and a generalized linear model (at the gene level). The main goal of this method is to reduce the high rates of type I errors reported in previous studies [17], i.e., false negatives. Abedalrhman and Rueda [28] presented the Zseq tool, indicating the importance of a pre-processing step in high-throughput sequencing data analysis. More specificity, Zseq is focused on the improvement in assembly of transcripts, evaluating the results of DEGs with different pre-processes. On the other hand, other approaches have been concerned with evaluating other perspectives such as the number of biological replications required for a RNA-Seq experiment and the most suitable tools for the analysis of differential expression based on the number of a experiment replicates [29]. A comprehensive and systematic analysis of the RNA-seq data from different perspectives presented by Sahraeian *et. al* (2017) can contribute significantly in addition to expression analyses from RNA-Seq data previously produced [30].

Differently from these studies, we evaluated the impact of the mapping methodology on the results of differential gene expression analysis. We also assess the methodologies of DEGs analysis through a different perspective, not only indicating the better methodologies. Previous studies and its results were presented, indicating that DEGs analysis are influenced by many factors such as preparation of libraries and the structure of the experiment. In this context, we analyzed the influence of essential steps in DEGs identification with RNA-Seq data and developed a software that allows to obtain the results of the main DEGs identification methodologies. The comparative study among the six mappers, including one pseudo-alignment and one quasi-mapping tool commonly used in differential expression studies led to identify the importance of this step in the analysis and identification of DEGs. A gold standard qRT-PCR data was also adopted in order to evaluate the accuracy of DEG identification tools and indicate those with high reliability on its results. Another contribution of this work was the evaluation of integrated results from DEGs identification methods, our tool allows to perform the consensus of five different methods of differential expression analysis, as a result genes indicated as differentially expressed have more reliability and accuracy.

In this study, we present an extended review of the main methodologies for differential gene expression analysis with RNA-Seq data, evaluating the impact of the mapping and quantification methodologies. For this study we adopted the mapping softwares Bowtie2 [31], TopHat [32], BWA [33] and STAR [34]. For other approaches, such as pseudo-alignment and quasi-mapping we adopted kallisto [35] and Salmon [36]. We analyzed differential expression analysis software that represents the state of the art in this field, such as baySeq [11], DESeq [12], DESeq2 [37], EBSeq [17], edgeR [13], limma+voom [38], NOIseq [15, 39] and SAMseq [16]. The mapping results were used as input for some differential expression analysis software tools, and its results were compared with qRT-PCR [40], thereby verifying the accuracy of each software associated with different mappers. The results indicate that NOIseq [15, 39], limma+voom [38] and DESeq2 [37], are the most balanced softwares by considering the precision, accuracy and sensitivity. We evaluated the results from individual and integrated ways, among different methodologies. The results indicate that a group of software can produce together high precision and accuracy than individual solutions. Finally, this work still presents as contribution a software tool easily applicable to different experiments for differential gene expression analysis. The software tool presents an integrated execution with mapping, mapping count (if necessary) and quantification of expression levels, indicating characteristics of the adopted methods with respect to their properties and accuracy when identifying DEGs.

## Materials and methods

### Datasets

This work adopted a real data set produced for the Microarray Quality Control (MAQC) project [10, 40]. The data set was obtained using the Illumina’s Genome Analyzer II. The experiment analyzed two biological samples: RNA from Ambion’s human brain and Stratagene’s human universal reference RNA, which we will refer in this work as Brain and UHR sets respectively [10]. We used only the Brain and UHR samples that used PhiX Control. The data set is available at the NCBI Short-Read Archive (SRA), under accession SRA010153. The reads were mapped against the human genome/transcriptome, version 19 (GRCh37.p13).

As part of the MAQC project, approximately a thousand genes were analyzed by qRT-PCR [41]. The qRT-PCR data is available at *Gene Expression Omnibus*, access: GSE5350, platform GPL4097 [40]. The Ambion human brain and Stratagene universal human samples were also adopted as biological references in this experiment. We considered the qRT-PCR data as a gold standard for the evaluation of the DEGs identification methods.

The conversion from the annotation for the RNA-Seq data (ENSEMBL) to the qRT-PCR data was performed by the on line tool bioDBnet [42], which excludes duplicate IDs or synonymous. The conversion generated a list of 997 unique qRT-PCR genes. For detailed information on the qRT-PCR gene listing see S1 Table.

### Sequence alignment and gene counts

The adopted RNA-Seq data set was mapped in the human genome/ transcriptome (hg19), with the annotation file of the same version, both from the GENCODE project [43]. The conversion from transcriptome to genome annotation was performed by the R package txImport [44]. For the mapping and quantification, various approaches were used: spliced read aligner, unspliced read aligners, pseudo-alignment and quasi-mapping. For the spliced read aligner approach the TopHat software was used (v.2.1.0) [18], which applies the exon-first methodology. For the unsigned read aligner approach two mapping softwares were used, BWA (v.0.7.12-r1039) [33] and Bowtie (v.2.2.6) [31], which apply the Burrows-Wheeler transform. For pseudo-alignment approach kallisto software was used (v.0.43.1) [35]. For quasi-mapping approach Salmon (v0.8.2) software was used [36]. For the mapping execution, the default parameters of each software were adopted. Table 1 presents the adopted mappers.

**Table 1:** The adopted mapping methods.

Name	Version	Mapping	Reference
Bowtie	2.2.6	Unspliced read aligner	[31]
BWA	0.7.12-r1039	Unspliced read aligner	[33]
TopHat	2.10	Spliced read aligner	[18]
STAR	2.5.3	Spliced read aligner	[34]
kallisto	0.43.1	pseudo-alignment	[35]
Salmon	0.8.2	pseudo-alignment	[36]

The HTSeq software (v.0.6.0) [12] was adopted to generate the count matrix, with default parameters. The adopted annotation file to generate the count matrix was the same one used in the mapping.

**Fig 1:**
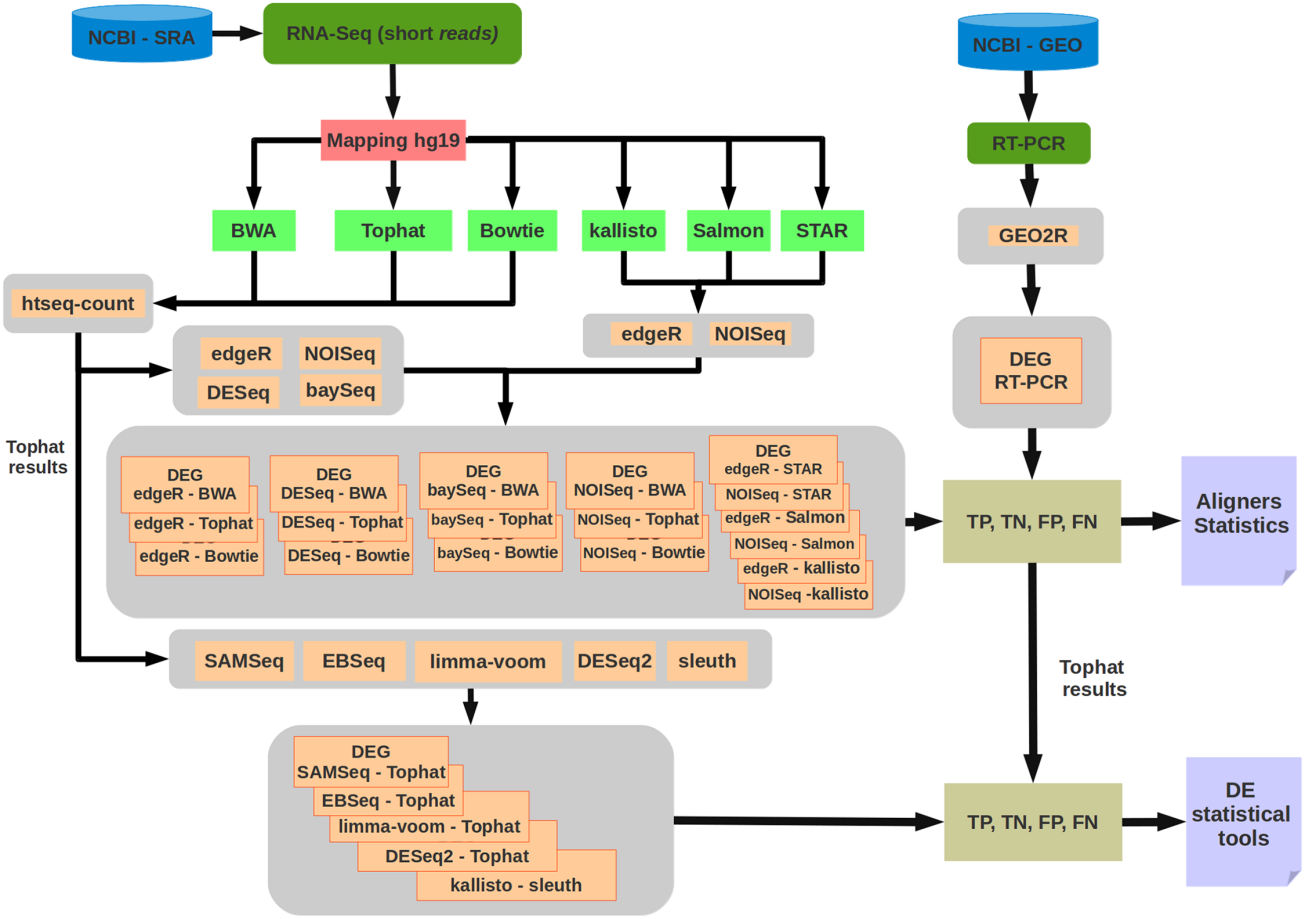
Overview of the pipeline presented in this work. The adopted biological samples to generate the qRT-PCR data were the same as those used to generate the RNA-Seq data.

Fig 1 presents an overview of the present work. The RNA-Seq dataset (denoted as “NCBI-SRA” in Fig 1) was mapped by each adopted method to the human genome (hg19), thereby obtaining counting matrices. The matrices were used as input for the adopted differential expression methods. In order to evaluate the impact of the mapping software on the DEGs identification, we analyzed four differential expression software using the six generated counting matrices. For Salmon, STAR and kallisto we analyzed two differential expression software.

The mapper performance were obtained by considering the following DEG identification methods: edgeR, DESeq, baySeq and NOIseq, to Tophat, Bowtie2 and BWA, to analyze Salmon, STAR and kallisto were performed edgeR and NOISeq. DESeq and baySeq can be run only with count data (Fig 1).

### Differential expression

In this work we compared eight methods for the DEGs or transcripts identification. When applying each software we focus on the most used approaches. Thus, we follow the guidelines available in the manual, applying the default parameters, including the standardization methodology of each software. All differential expression analysis were performed using the same counting matrix, generated by HTSeq.

For each evaluated mapper a counting matrix, or abundance matrix was generated, in this way the mapper tools were evaluated for DEG methods. Table 2 presents a summary of the adopted DEG identification methods and its properties.

**Table 2:** Adopted methods for DEGs identification.

Name	Version	Normalization	Reference
baySeq	2.4.1	Scaling factors (quantile/ TMM/ total)	[11]
DESeq	1.22.1	DESeq size factors	[12]
EBSeq	1.12.0	DESeq median normalization	[17]
edgeR	3.12.1	TMM/ Upper quartile / RLE / None (all scaling factors are set to be one)	[13]
limma+voom	3.26.9	TMM	[38]
NOIseq	2.14.1	RPKM / TMM / Upper quartile	[15, 39]
SAMseq (samr)	2.0	Based on the read count mean over the null features of data set.	[16]
DESeq2	1.10.1	DESeq size factors	[37]
sleuth	0.29.0	DESeq size factors (with slight modifications)	[35]

RNA-Seq data were mapped using BWA, TopHat, Bowtie and STAR mappers. The quantification was obtained from Salmon and kallisto tools. The counting table of each mapper was used as input for the DEG identification methods (edgeR, DESeq, baySeq and NOISeq), thus generating lists of DEGs for each DEG identification method which different mapper. Results of Salmon, STAR and kallisto was used as input for edgeR and NOISeq. The results were compared with qRT-PCR (gold standard), allowing to evaluate if the mapping influences the performance of DEGs detection. The EBSeq, SAMSeq and limma+voom, DESeq2 and sleuth methods were added to the study for individual evaluation of DEG identification tools, using only the mapping results of the TopHat mapper. Exceptionally sleuth received quantification output from the kallisto tool, as indicated in its user guide. Fig 1 presents an overview of the pipeline presented in this work.

The DEGs identified by the adopted methods (with TopHat mapper) are used to evaluate the performance statistics presented in the Results Section.

- **baySeq** [11]: Uses the Bayesian empirical approach to estimate a posteriori probability of each set of models, which defines differential expression patterns for each tuple.
- **DESeq** [12]: Based on a negative binomial distribution, with variance and mean bound by local regression.
- **EBSeq** [17]: Developed with the main objective of identifying differentially expressed isoforms, it is also robust in the identification of DEGs. It is similar to baySeq [11], which applies the Bayesian empirical approach.
- **edgeR** [13]: A Poisson super dispersion model is used to account for technical and biological variation. Apply the Bayesian empirical method to moderate the degree of over dispersion against transcripts.
- **limma+voom** [38]: Based on the linear model and originally developed to analyze data from microarray and currently extended for RNA-Seq analysis. The limma user guide recommends the use of the TMM normalization of the edgeR package associated with the use of the voom conversion, which essentially transforms the normalized counts to logarithms base 2 and estimates the mean-variance relation to determine the weight of each observation made initially by a linear model [45].
- **NOIseq** [15, 39]: Adaptive to the data and non-parametric, empirically models the noise in the counting data and allows the data analysis without replication.
- **SAMseq**: [16]: Non-parametric method with re-sampling for sequencing counts with different depths. It can be applied to data with quantitative results, two-class, or multiple-class.
- **DESeq2** [37]: DESeq2 firstly build a model with observed counts. Secondly, it fits using the same method from the original DESeq, or fit in two steps: find the value of the parameter that makes the likelihood largest, which is called maximum likelihood estimation. Then, it takes all the gene values and move these values towards a average value. DESeq2 uses Bayes theorem to guides the amount of movement for each gene: if the information for the gene is low, its value is moved close to the average, if the information for the gene is high, its value is moved very little. Thus, the moved values are useful to evaluate different sets of genes as well as to apply a threshold;
- **sleuth** [35]: The sleuth workflow begins with a filtering of low abundance transcripts, followed by the application of two normalizations and then parameter estimation for the model of each transcript. This enables the regularization of the biological variance contributing to transcript abundance variance across samples, and finally to an overall total variance estimate for each transcript.

## Results and discussion

### Read mapping in reference genome

In order to evaluate the mapping methods, the human genome described in subsection *Datasets*. To evaluate the impact of the genomic mapping tool on DEG analysis, all the mapping software with default parameters were adopted. The counting matrix of each mapping was generated by the HTSeq package [12], through the htseq-count function, using the genome annotation file and default parameters. The counting matrix of each mapping tool was used as input for the DEG detection methods. Regarding qRT-PCR data, the DEG was unidentified by adopting the GEO2R tool from default method (Benjamini & Hochberg). It were considered as DEGs only the transcripts with *log*_2_*FC* ≥ ±2 and *P*–*value* ≤ 0.05. The complete list of DEGs is available in S1 Table.

**Fig 2:**
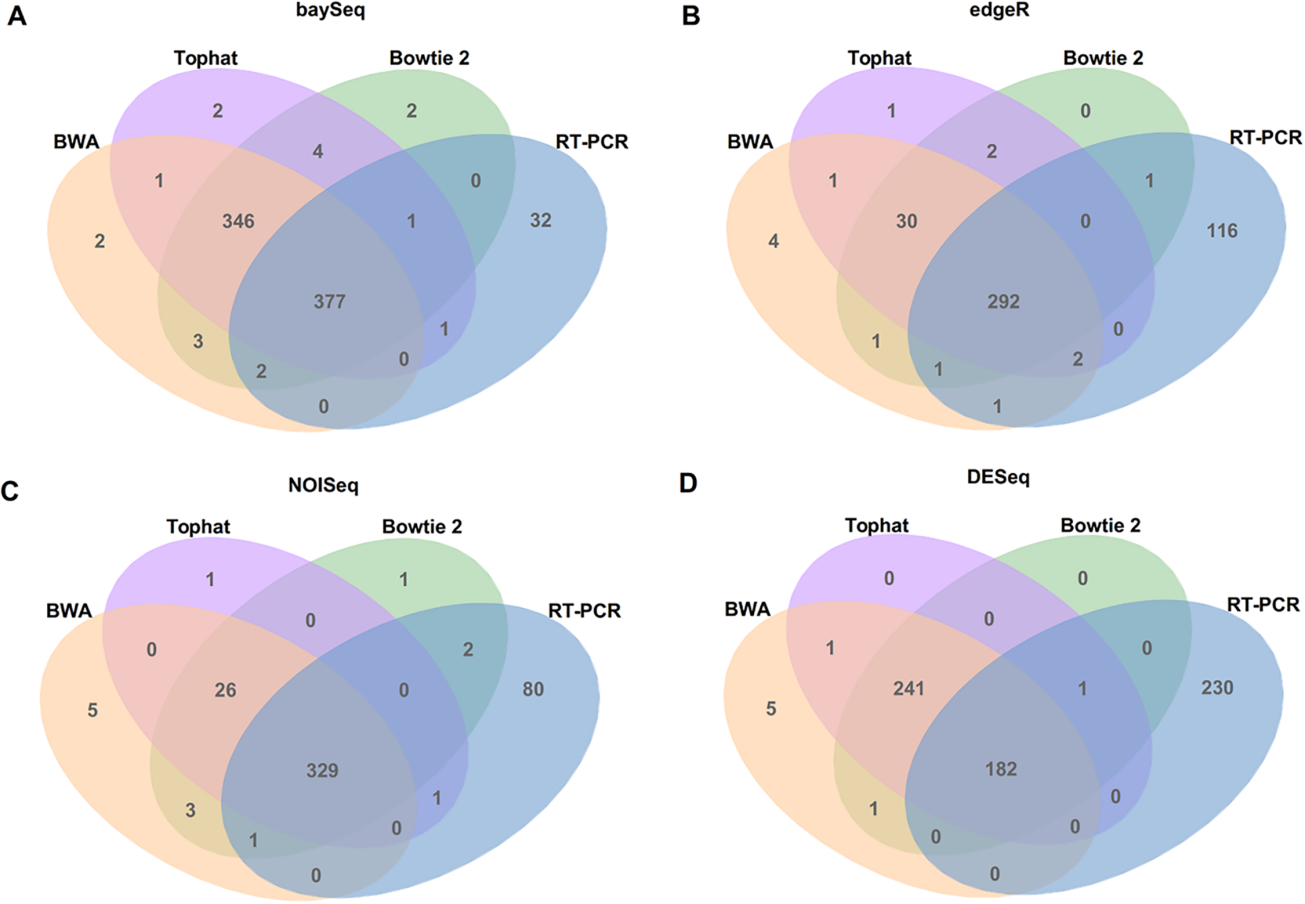
Comparison of identified DEGs from different expression analysis tools, associated to distinct RNA-Seq mapping methods compared to qRT-PCR. (A) Venn diagram comparing identified DEGs by the baySeq tool with BWA, TopHat, Bowtie and qRT-PCR mappers. (B) Venn diagram comparing identified DEGs by the edgeR tool with BWA, TopHat, Bowtie and qRT-PCR mappers. (C) Venn diagram comparing identified DEGs by the NOIseq with BWA, TopHat, Bowtie and qRT-PCR mappers. (D) Venn diagram comparing identified DEGs by the DESeq with BWA, TopHat, Bowtie and qRT-PCR mappers.

We compared the identified DEGs in RNA-Seq (baySeq, edgeR, DESeq and NOIseq) against the DEGs from qRT-PCR. It is possible to observe in Fig 2 and in Table 3 that DEGs are concentrate on the intersections between the mappers, showing that the methods maintain the identification behavior even with the change of the mapping methodology.

**Table 3:** Comparison of the number of identified DEGs from different expression analysis tools, associated to different RNA-Seq mapping methods compared to qRT-PCR. DEGs indicated by the edgeR and NOISeq tool using data from different mappers. qRT-PCR row indicates the amount of correctly labeled DEGs.

**edgeR**							
	**Tophat**	**Bowtie**	**BWA**	**STAR**	**Salmon**	**kallisto**	**qRT-PCR**
**Tophat**	328						
**Bowtie**	324	327					
**BWA**	325	324	332				
**STAR**	319	319	319	370			
**Salmon**	299	298	300	304	333		
**kallisto**	302	303	307	306	325	354	
**qRT-PCR**	294	294	296	338	294	304	413
**NOISeq**							
	**Tophat**	**Bowtie**	**BWA**	**STAR**	**Salmon**	**kallisto**	**qRT-PCR**
**Tophat**	357						
**Bowtie**	286	310					
**BWA**	284	306	309				
**STAR**	295	293	292	303			
**Salmon**	284	295	295	284	332		
**kallisto**	282	292	292	283	323	323	
**qRT-PCR**	330	275	274	281	276	274	413

In this way, we observed that the impact of the mappers on the final results is minimal. In Fig 2A and 2C, it is possible to observe that the number of DEGs correctly identified (in agreement with qRT-PCR) is more related to the methodology of DEG identification than to the adopted mapper. The baySeq and NOIseq methodologies obtained a low amount of unidentified DEGs, and this amount was not changed with different mappers.

In order to evaluate methods that do not use mapping, but other strategies to quantifying reads, we compared edgeR and NOISeq results using Salmon and STAR to quantification of mapped genes. Table 3 presents the number of DEGs identification from different RNA-Seq mapping methods. It was considered only NOISeq and edgeR because baySeq and DESeq are not able to receive inputs different of integers values. Once again the result indicates that differential expression analysis is more influenced by the methodology of DEGs identification than the adopted methodology of mapping or quantification of reads.

S2 Table presents more details about the performance of each DEGs identification method with different mappers.

### DEG identification methods

As presented in the previous section, the impact of the mappers on expression analyses is minimal. In this way, all following analysis were developed considering only the TopHat mapping results. At this step of the present work, we analyze the results of the following software tools: limma+voom [38], EBSeq [17], SAMseq [15], DESeq2 [37] and sleuth [35]. For more details regarding the evaluated tools see the subsection *Differential Expression*.

We compared the genes indicated as differentially expressed by the nine tools and the DEGs indicated by qRT-PCR. The softwares were performed as defined in each manual, and the genes listed by the tools were considered as differentially expressed, through the limit indicated by the manual of each tool. The performance of the adopted DEG identification methods were evaluated based on the match between each method results and the qRT-PCR. Table 4 presents the performance of each adopted method.

**Table 4:** Performance of the DEGs software tools regarding the qRT-PCR results. Performance measures adopted: TPR (True Positive Rate), SPC (Specificity), PPV (Positive Predict Value), ACC (Accuracy) and *F*_1_ measure [46, 47].

Tool	TPR	SPC	PPV	ACC	*F*_1_ measure
edgeR	0.71	0.94	0.90	0.85	0.79
baySeq	0.92	0.40	0.52	0.61	0.66
DESeq	0.44	0.59	0.43	0.53	0.44
NOIseq	**0.80**	**0.95**	**0.92**	**0.89**	**0.86**
SAMseq	0.44	0.52	0.39	0.49	0.42
limma+voom	**0.81**	**0.93**	**0.89**	**0.88**	**0.85**
EBSeq	0.68	0.55	0.52	0.60	0.59
DESeq2	**0.84**	**0.95**	**0.92**	**0.90**	**0.88**
sleuth	0.77	0.54	0.54	0.63	0.64

It is possible to notice that the EBSeq, SAMseq and DESeq methods, although using different approaches for DEG identification have similar behavior, presenting low TPR (True Positive Rate) and low ACC (Accuracy). The performance of the DESeq can be justified by the fact that the tool obtains better results with small samples (two samples per condition), as presented in [22]. The results of the SAMseq are very influenced by the sample size and the number of replicates. The SAMseq is able to ranking the most relevant DEGs, however its results produce many false positives [22, 25].

The NOIseq, DESeq2 and limma+voom methods performed well, with high TPR and ACC rates. The limma+voom tool had already been pointed out in previous works as one of the better results in the DEG ranking and for analyses with more than two samples [22]. NOIseq and DESeq2 tools showed consistent results, indicating these methods are suitable for experiments with a large number of samples and an annotated genome.

### Integration of DEG identification methods

The individual evaluation of DEG identification methods indicate clearly that each method led to distinct results. Moreover, some methodologies have better results with a greater amount of samples, while others present variations on its results influenced by other characteristics, such as sequencing depth and outliers with abnormally high counts.

In order to verify the compatibility between the individual results of each DEG identification method and to identify possible improvement in performance, we evaluated the results by integrating the adopted methodologies in this work. We evaluated the performance among the results with the integration of nine to one methods, so that for each gene identified as differentially expressed by *x* methods, where *x* is number of methods that have identified each DEG. The results of each combination of DEG identification methods was compared to the gold standard from qRT-PCR.

**Fig 3:**
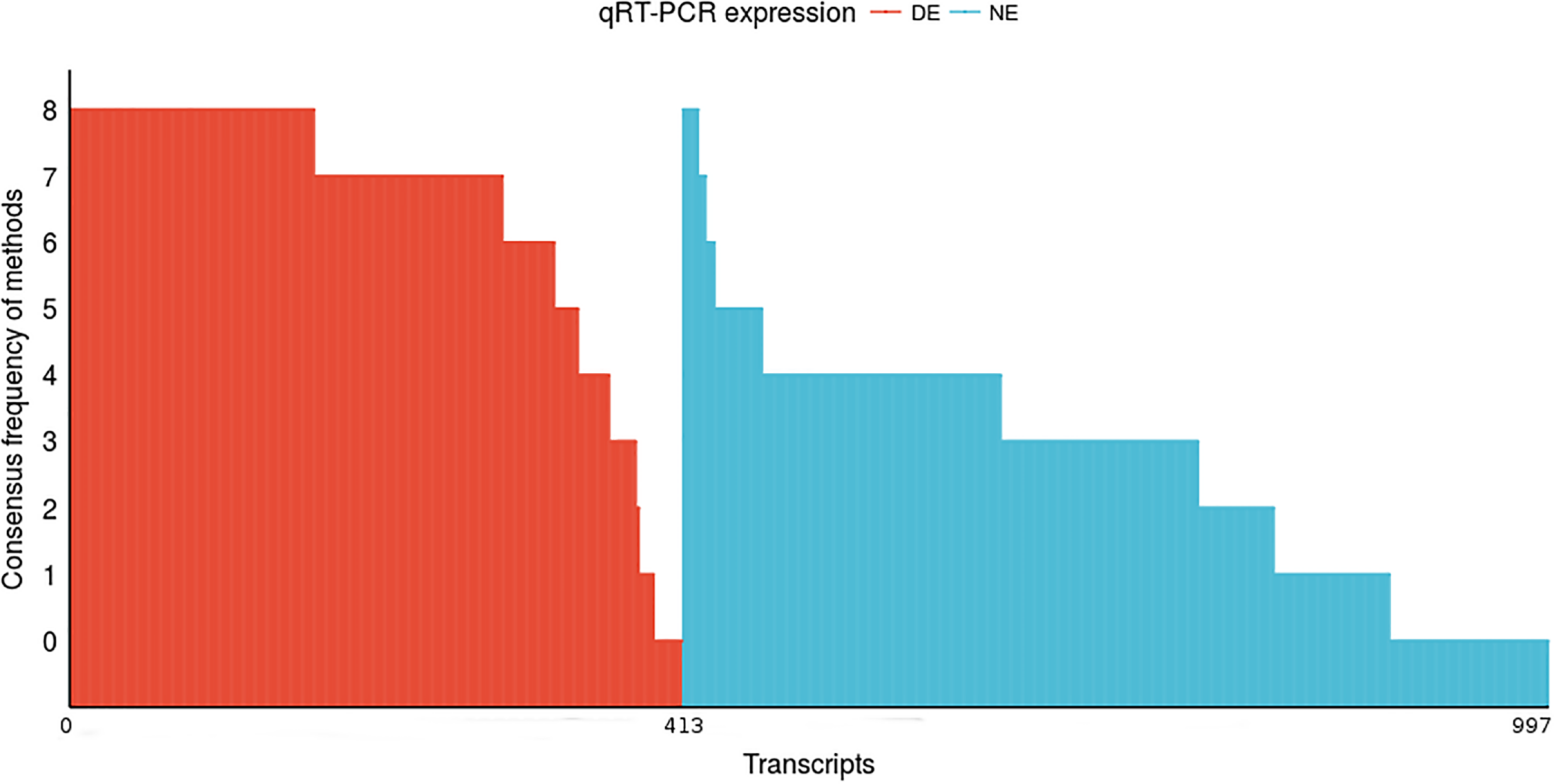
Histogram from DEGs identification methods integration. The red bars indicate the DEGs identified as differentially expressed (True Positives). The blue bars indicate the not differentially expressed transcripts identified as DEGs from methods (False Positives). The Y axis indicates the amount of tools that identified correctly the transcripts as differentially expressed or not. The first row (bars with 0 in Y axis) indicate DEGs and not differentially expressed genes from qRT-PCR (gold standard) with 413 DEGs and 584 not differentially expressed transcripts, totaling 997 genes analyzed. There are no performance values for nine tools, since there was no convergence of the results with transcripts indicated by nine methods as DEG.

In order to evaluate the performance of the DEG integration methods, we verified the combination of methods that performs better. Fig 3 present the integration from one to nine DEG identification methods. It is possible to notice that there were no congruence of differentially expressed transcripts from the integration of nine methods. From the nine evaluated methods, the frequency of eight simultaneous indications occurs for 165 transcripts also indicated by qRT-PCR as differentially expressed. However, when observing the number of DEGs indicated by qRT-PCR, it is possible to observe that integration of eight methods fails to identify a large number of genes indicated by qRT-PCR.

To identify the combination of methods that has a more effective DEGs indication and, with the least amount of errors, we evaluated the DEGs identification performance for each subset of methods: nine, eight, seven, six, five, four, three, two and one. The performance results of each subset is presented in Table 5.

**Table 5:** Performance of each subset of DEGs identification methods.

Number of indications	TPR	SPC	PPV	ACC	*F*_1_ measure	FPR
1	0.95	0.18	0.95	0.62	0.61	0.82
2	0.93	0.32	0.93	0.74	0.64	0.68
3	0.92	0.40	0.92	0.88	0.67	0.60
4	0.88	0.63	0.88	0.89	0.73	0.37
5	**0.83**	**0.91**	**0.83**	**0.86**	**0.85**	**0.09**
6	0.79	0.96	0.79	0.74	0.86	0.04
7	0.71	0.97	0.71	0.59	0.81	0.03
8	0.40	0.98	0.40	0.00	0.56	0.02

The subsets did not have a selection of specific methods, only the frequency of indications was observed. There are no performance values for nine tools, since there was no convergence of the results with transcripts indicated by nine methods as DEG.

As expected, the performance of each subset indicate that to consider more methods together tends to improve the accuracy and to reduce the error rates. As reported in the context of the inference of gene networks, the collective knowledge or data integration can produce better results than individual results [48, 49]. Based on this principle, we identified that the integration of five methods can obtain higher TPR and SPC values than any other tested subset.

**Fig 4:**
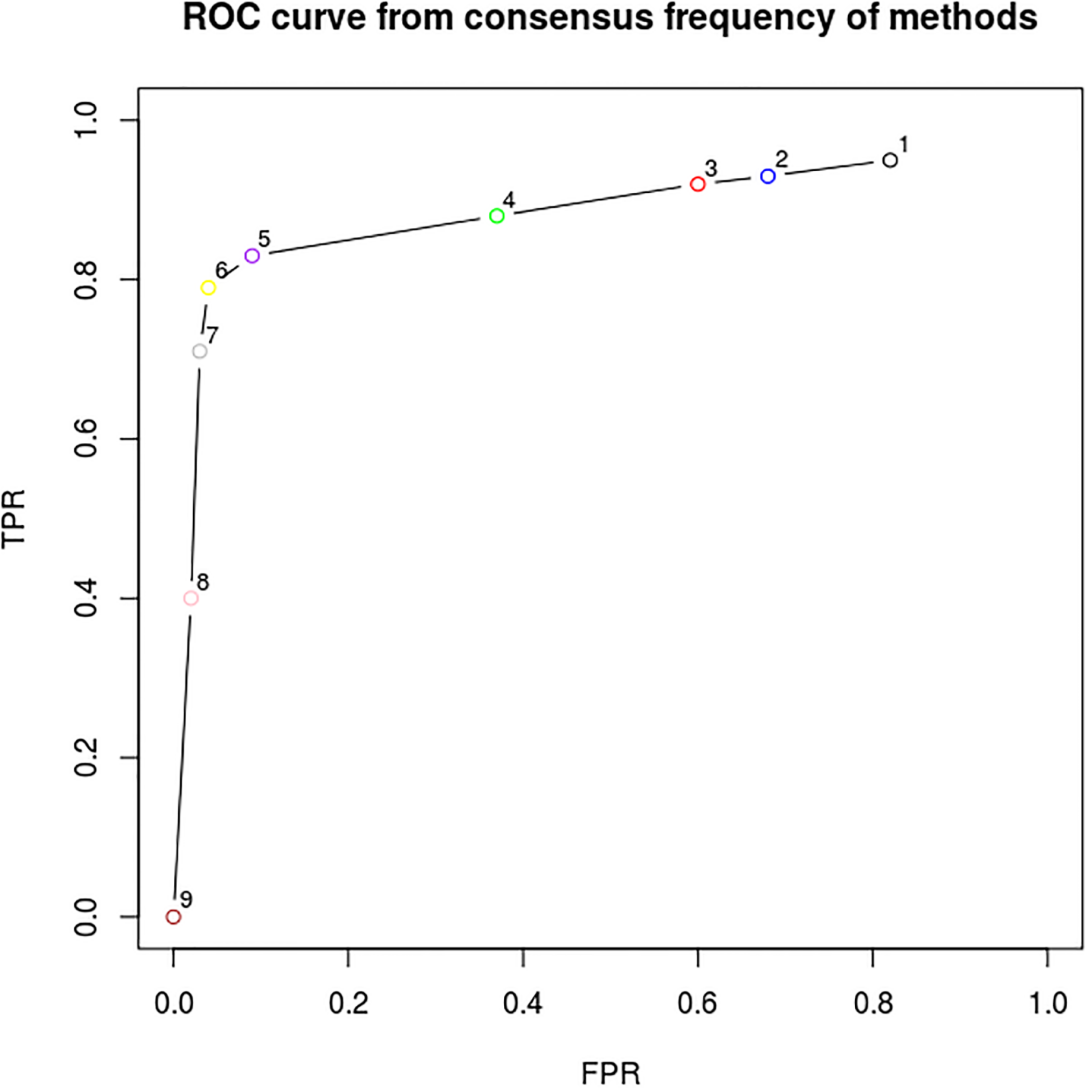
ROC curve from integration of DEG identification methods. Each point indicate the performance of the best subset regarding the adopted qRT-PCR.

In order to identify the best combination of DEG identification methods of each cardinality (1, 2, …, 9), we adopt the ROC (Receiver operating characteristic) curve [50], a standard pattern recognition tool. Fig 4 present the better combination of DEG identification methods consensus. It is possible to notice that combination of five methods presents the most efficient solutions among all the tested combination. The consensus among six methods led to a slight improvement in FPR, however also present a decline in TPR.

**Fig 5:**
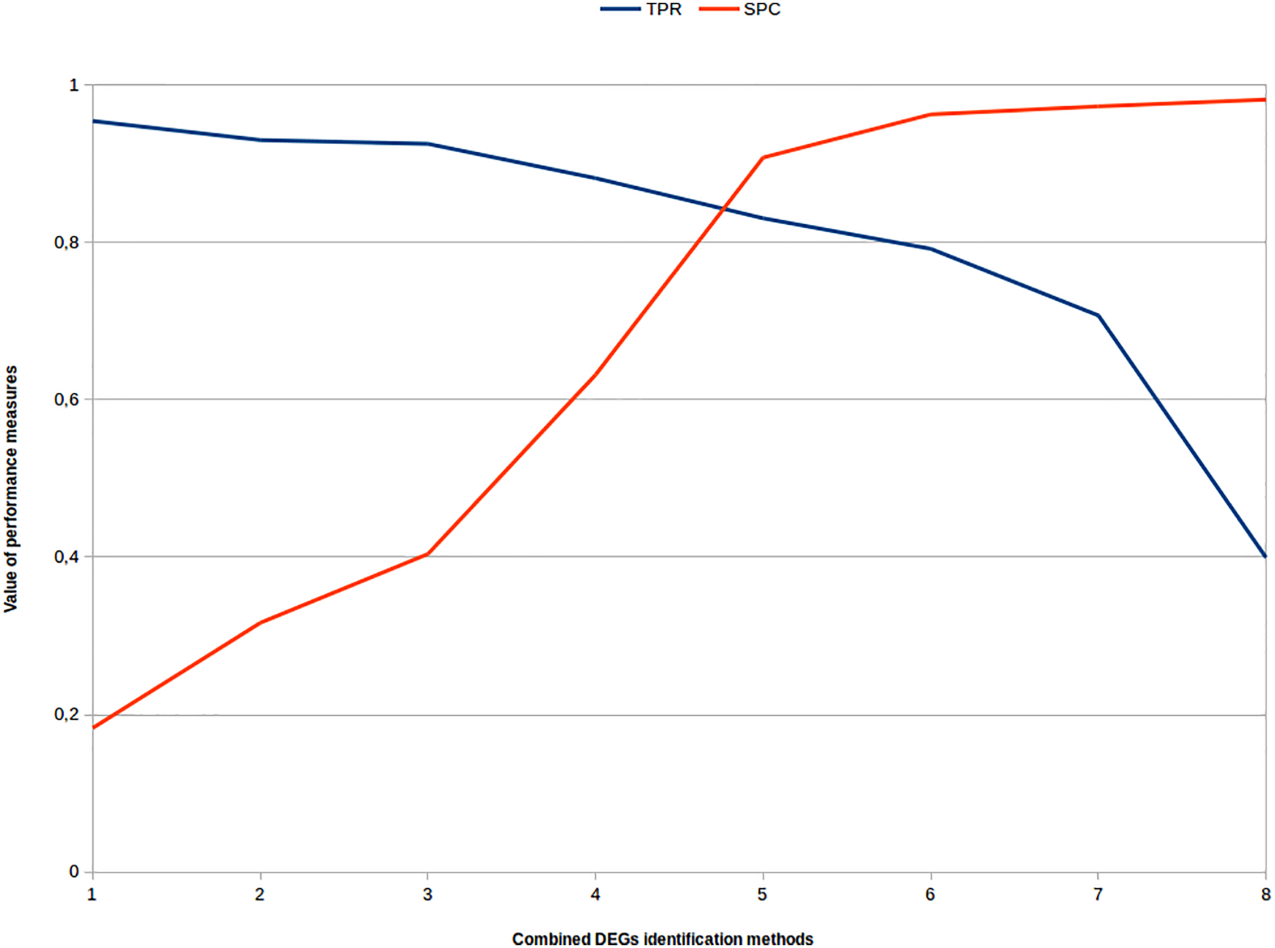
Projection curves of TPR and SPC. Projection curves of TPR and SPC values when combining DEGs identification methods. The X axis is the quantity of combined DEGs identification methods. The Y axis is the evolution of TPR and SPC values regarding the adopted qRT-PCR.

The consensus of five DEGs identification methods presented the best integrated result with higher SPC and TPR values that lead to results with high accuracy. Fig 5 presents the evolution of the TPR and SPC values related by increasing the integration of DEGs identification methods.

The inclusion of methods brought considerable gains in specificity (SPC), however from integration of six methods, TPR values goes through a considerable decline. This result indicated the default value for the software available at https://github.com/costasilvati/consexpression, in which the user can choose between executing the methodology with the default consensus (five methods), thus obtaining the best balance between SPC and TPR. Another possibility for the application of this methodology is to change the number of methods that define the consensus for the desired quantity of methods, taking into account the cost of the change, for the other performance measures, as well as to use only one of the adopted methods in this work.

Table 6 presents an overview of the groups of methods that correctly identified the DEG according to qRT-PCR. Regarding the 413 DEGs from qRT-PCR, 19 of them were not identified by any method. It is possible to observe that when considering one indication as differentially expressed (accepting any tool) it was not possible to reach the 413 genes indicated by qRT-PCR.

**Table 6:** Relation between True Positives (TP) and aggregate results from number of methods.

${\text{N}}^{\underline{\text{o}}}$ methods	TP
0	19
1	394
2	384
3	382
4	364
5	343
6	327
7	292
8	165
9	0

Regarding the 413 genes identified as differentially expressed (DE) by qRT-PCR, we grouped by number of methods that indicated DEGs correctly.

In order to define which group of methods has the best consensus, it is important to evaluate how each method behaves in the aggregate results, especially in the group of five indications. Table 7 presents the frequency of each method in the aggregate results. Comparing the results presented in Tables 7 and 6, we can observe that 343 DEGs pointed out by consensus of five methodologies, the methods that most correctly pointed out (almost all indications) were baySeq [11], DESeq2 [37], limma+voom [38] and NOISeq [15, 39], respectively.

**Table 7:** Number of correctly identified DEGs from each method considering the aggregate results (consensus).

${\text{N}}^{\underline{\text{o}}}$ of methods	edgeR	baySeq	DESeq	NOISeq	SAMSeq	limma+voom	EBSeq	DESeq2	sleuth
9	0	0	0	0	0	0	0	0	0
8	165	165	90	165	75	165	165	165	165
7	248	292	147	292	145	287	236	292	270
6	271	327	158	326	160	316	252	326	283
5	280	343	167	329	164	331	259	335	291
4	285	363	176	330	174	334	273	340	308
3	293	378	180	330	181	335	281	348	311
2	293	379	181	330	182	335	282	348	311
1	294	379	183	330	182	335	282	348	318

Regarding the consensus of five methods, the baySeq method indicates all DEGs presented in the five consensus results. The DESeq2 indicate 97.6%, limma+voom methods indicate 96.5% of them, and NOISeq indicates 95.9%. For the analysis with baySeq, it is necessary to define a collection of models and each model is a subdivision of the samples into groups, the samples in the same group are assumed to share the same parameters of the underlying distribution. In the DESeq2 method, a model is created to the counts observed, this model is fit using Bayes theorem to guide the movement by each gene. In the NOISeq method, the transcript is differentially expressed if the ratio of *log*_2_ between two conditions and the value of the difference between the two corresponding conditions are likely to be higher than a noise. The noise distribution is obtained by comparing all pairs of repetitions within the same condition. In the limma+voom method, the read counts are converted into *log*_2_ of counts per million (logCPM) and the mean variance ratio is modeled with precision weights.

In summary, the baySeq method tends to higher FP values, as presented in Table 4 which indicates 100% of the DEGs consensus of five methods. The parameter sharing of the sample groups of this methodology, mitigates the variation of the genes of the same group, thus leading to a greater probability of correctness for the methodology. On the other hand, NOISeq, DESeq2 and limma+voom methods perform in a balanced way regarding its relation to the correctly DEGs identification allowing a high reliability of the results, which justifies only the ≃3.8% DEGs not identified by them and identified by qRT-PCR. Regarding edgeR results, we can verify that its TPR tends to a lower reliability, presenting 81.3% of correct identification of the DEGs indicated by qRT-PCR.

## Conclusion

This work presents an extended review regarding methods for the identification of differentially expressed genes (DEGs) or transcripts. We evaluated the influence of six mapping methods, including one pseudo-alignment and one quasi-mapping, nine main methods for the DEGs identification and the integration of these methods in order to produce a consensus from their results. The evaluation of the adopted methods was performed by comparing the respective results from a reference qRT-PCR for the same tested transcripts.

We have identified that the impact of the mapping tool on the final results is minimal, indicating the DEGs identification method is the main choice for differential expression analysis in RNA-Seq data.

We did not identify among the evaluated methods a tool that obtained optimum results in all performance measures, for the evaluated experimental conditions. The NOIseq, DESeq2 and limma+vomm methods present the best individual results with 95%, 95% and 93% of Specificity and 80%, 84% and 81% of True Positive Rate, respectively.

Regarding the integration of DEG identification methods, we identified that the combination of five methods improves the sensitivity of identification and provides more reliable results. The five methods used integrated produced results with 91% of Specificity and 83% of True Positive Rate, thus indicating the consensus of five methods present better balance than individual solutions.

Finally, this study also contribute to present a freely available software at https://github.com/costasilvati/consexpression, which implements the presented analysis and can be easily used in order to replicate this work, as well as to analyze other RNA-Seq data sources.

## Supporting information

S1 Table qRT-PCR analysis. Differentially expressed genes indicated by qRT-PCR.
(PDF)

S2 Table Mapping analysis. Performance of each DEGs identification method with different mappers.
(PDF)
