# Supplementary material for: RNA-Seq differential expression analysis: An extended review and a software tool
Source: PLoS One. 2017 Dec 21;12(12):e0190152. doi: 10.1371/journal.pone.0190152 (PMC5739479; doi:10.1371/journal.pone.0190152)
Supplement: S1 Table — Differentially expressed genes indicated by qRT-PCR. (PDF) [file pone.0190152.s001.pdf]

Differential expressed list to GSE5350 - GPL4097 dataset:

Table 1: Evaluation of DEGs for qRT-PCR. Differentially super-expressed genes are indicated with 1, the sub-expressed with -1, and not expressed genes with 0.

**Gene**	**Expression**
ENSG00000113924	-1
ENSG00000112658	0
ENSG00000129159	1
ENSG00000049283	0
ENSG00000018625	1
ENSG00000086570	1
ENSG00000006116	1
ENSG00000108821	-1
ENSG00000016602	1
ENSG00000121570	0
ENSG00000125630	0
ENSG00000165704	0
ENSG00000101746	1
ENSG00000112977	-1
ENSG00000135636	0
ENSG00000117724	-1
ENSG00000166479	0
ENSG00000109158	1
ENSG00000166510	0
ENSG00000221829	-1
ENSG00000168394	0
ENSG00000133169	1
ENSG00000160194	0
ENSG00000055332	0
ENSG00000171824	0
ENSG00000106462	-1
ENSG00000006071	1
ENSG00000160957	0
ENSG00000124491	1
ENSG00000089116	1
ENSG00000066739	0
ENSG00000065485	-1
ENSG00000003756	0
ENSG00000168309	1
ENSG00000130643	1
ENSG00000108984	0
ENSG00000079257	-1
ENSG00000173208	1
ENSG00000148688	0
ENSG00000151422	0
ENSG00000154485	1

ENSG00000108651	0
ENSG00000104964	0
ENSG00000088986	0
ENSG00000086848	0
ENSG00000182551	0
ENSG00000068323	0
ENSG00000130193	0
ENSG00000073734	0
ENSG00000166598	0
ENSG00000068097	0
ENSG00000196611	-1
ENSG00000086696	-1
ENSG00000136527	0
ENSG00000170425	0
ENSG00000113068	0
ENSG00000103769	0
ENSG00000134061	0
ENSG00000168040	0
ENSG00000126838	0
ENSG00000005189	-1
ENSG00000106571	0
ENSG00000065457	0
ENSG00000198851	-1
ENSG00000101442	0
ENSG00000112118	-1
ENSG00000137801	-1
ENSG00000166396	-1
ENSG00000260027	-1
ENSG00000171853	0
ENSG00000025708	0
ENSG00000137502	0
ENSG00000163586	-1
ENSG00000167157	-1
ENSG00000137757	0
ENSG00000033122	1
ENSG00000144843	0
ENSG00000103222	-1
ENSG00000127191	0
ENSG00000132849	0
ENSG00000104447	0
ENSG00000136536	0
ENSG00000171241	-1
ENSG00000168259	0
ENSG00000136807	0
ENSG00000011009	0
ENSG00000056736	0
ENSG00000168148	0

ENSG00000135114	0
ENSG00000131914	-1
ENSG00000134240	-1
ENSG00000129244	1
ENSG00000151470	0
ENSG00000182578	1
ENSG00000240972	0
ENSG00000044524	0
ENSG00000068383	1
ENSG00000145545	0
ENSG00000084731	1
ENSG00000102239	0
ENSG00000106004	-1
ENSG00000147140	0
ENSG00000106565	1
ENSG00000171793	-1
ENSG00000116560	0
ENSG00000106852	1
ENSG00000004059	0
ENSG00000187048	1
ENSG00000259494	0
ENSG00000184524	1
ENSG00000122729	0
ENSG00000112578	-1
ENSG00000157404	1
ENSG00000154146	1
ENSG00000104327	1
ENSG00000073598	0
ENSG00000110651	0
ENSG00000150526	-1
ENSG00000105639	0
ENSG00000125037	0
ENSG00000169393	-1
ENSG00000128606	-1
ENSG00000178591	0
ENSG00000127990	0
ENSG00000131264	0
ENSG00000106367	0
ENSG00000156006	0
ENSG00000093009	-1
ENSG00000144935	1
ENSG00000140505	0
ENSG00000163818	0
ENSG00000181222	0
ENSG00000156076	1
ENSG00000221946	1
ENSG00000268104	-1

ENSG00000121270	0
ENSG00000176124	0
ENSG00000170745	0
ENSG00000161981	0
ENSG00000089685	-1
ENSG00000080166	-1
ENSG00000167635	0
ENSG00000163536	1
ENSG00000169860	0
ENSG00000140538	1
ENSG00000066455	0
ENSG00000080986	-1
ENSG00000131236	0
ENSG00000157445	1
ENSG00000133962	0
ENSG00000148291	0
ENSG00000187323	1
ENSG00000180660	1
ENSG00000172172	0
ENSG00000104812	0
ENSG00000256683	0
ENSG00000103653	0
ENSG00000149548	-1
ENSG00000134245	1
ENSG00000172409	0
ENSG00000169432	0
ENSG00000237889	0
ENSG00000168385	0
ENSG00000166710	0
ENSG00000170925	0
ENSG00000077150	-1
ENSG00000049249	-1
ENSG00000239713	-1
ENSG00000160211	0
ENSG00000197408	0
ENSG00000158161	0
ENSG00000002587	1
ENSG00000117054	0
ENSG00000120669	0
ENSG00000171819	0
ENSG00000171711	0
ENSG00000152683	0
ENSG00000101638	1
ENSG00000118855	0
ENSG00000168002	0
ENSG00000100083	0
ENSG00000071553	0

ENSG00000124664	-1
ENSG00000178401	-1
ENSG00000063245	0
ENSG00000130816	-1
ENSG00000162174	0
ENSG00000090339	-1
ENSG00000084628	0
ENSG00000069011	-1
ENSG00000139291	0
ENSG00000138075	-1
ENSG00000138685	0
ENSG00000157890	1
ENSG00000079739	0
ENSG00000070814	0
ENSG00000114770	0
ENSG00000121940	0
ENSG00000188641	0
ENSG00000115009	-1
ENSG00000150961	0
ENSG00000119655	0
ENSG00000137204	0
ENSG00000162267	-1
ENSG00000115657	0
ENSG00000254685	0
ENSG00000198822	1
ENSG00000103254	0
ENSG00000109079	0
ENSG00000002933	1
ENSG00000140030	1
ENSG00000158497	0
ENSG00000100029	0
ENSG00000130021	0
ENSG00000075275	-1
ENSG00000165304	-1
ENSG00000144848	0
ENSG00000116754	0
ENSG00000115919	-1
ENSG00000136688	0
ENSG00000142619	-1
ENSG00000077522	1
ENSG00000128714	-1
ENSG00000109674	-1
ENSG00000077498	-1
ENSG00000179094	0
ENSG00000128340	-1
ENSG00000171564	-1
ENSG00000168124	0

ENSG00000134013	-1
ENSG00000170430	0
ENSG00000137563	0
ENSG00000130164	0
ENSG00000197299	-1
ENSG00000078140	0
ENSG00000146648	0
ENSG00000131910	-1
ENSG00000138798	0
ENSG00000178828	-1
ENSG00000115415	0
ENSG00000198816	0
ENSG00000228435	0
ENSG00000068971	1
ENSG00000112033	0
ENSG00000127774	0
ENSG00000135775	0
ENSG00000126353	0
ENSG00000160294	0
ENSG00000173698	-1
ENSG00000126581	0
ENSG00000181722	1
ENSG00000135346	-1
ENSG00000068120	0
ENSG00000183258	0
ENSG00000136457	1
ENSG00000164494	0
ENSG00000171223	0
ENSG00000129595	1
ENSG00000071991	1
ENSG00000110079	0
ENSG00000152291	0
ENSG00000105989	1
ENSG00000137877	1
ENSG00000123975	-1
ENSG00000167900	-1
ENSG00000214021	0
ENSG00000196329	1
ENSG00000106258	-1
ENSG00000156735	0
ENSG00000214193	0
ENSG00000064300	0
ENSG00000144366	-1
ENSG00000116171	0
ENSG00000134899	0
ENSG00000022355	1
ENSG00000004779	0

ENSG00000198380	0
ENSG00000099957	0
ENSG00000184674	0
ENSG00000120063	0
ENSG00000138061	0
ENSG00000122025	1
ENSG00000206075	-1
ENSG00000157601	0
ENSG00000169429	-1
ENSG00000165416	0
ENSG00000067704	0
ENSG00000135365	1
ENSG00000188257	-1
ENSG00000145386	-1
ENSG00000053501	0
ENSG00000198625	0
ENSG00000148516	0
ENSG00000002016	0
ENSG00000141510	-1
ENSG00000173992	0
ENSG00000135333	1
ENSG00000103351	0
ENSG00000159339	0
ENSG00000171365	0
ENSG00000106536	0
ENSG00000176783	0
ENSG00000134757	0
ENSG00000163295	-1
ENSG00000114648	0
ENSG00000014919	0
ENSG00000128294	0
ENSG00000175575	0
ENSG00000100985	0
ENSG00000059758	0
ENSG00000101470	-1
ENSG00000081800	0
ENSG00000196305	0
ENSG00000127980	0
ENSG00000100601	0
ENSG00000105248	0
ENSG00000184208	0
ENSG00000130045	-1
ENSG00000152284	-1
ENSG00000169398	0
ENSG00000167916	0
ENSG00000162374	1
ENSG00000198925	0

ENSG00000185245	0
ENSG00000118508	-1
ENSG00000120318	0
ENSG00000120756	0
ENSG00000151365	0
ENSG00000103876	-1
ENSG00000249961	0
ENSG00000154678	1
ENSG00000118217	0
ENSG00000114473	0
ENSG00000118245	0
ENSG00000127452	0
ENSG00000155090	-1
ENSG00000185432	0
ENSG00000133961	0
ENSG00000100604	1
ENSG00000064651	0
ENSG00000068137	0
ENSG00000108179	0
ENSG00000164106	1
ENSG00000107331	1
ENSG00000100479	-1
ENSG00000100028	0
ENSG00000092295	0
ENSG00000165029	0
ENSG00000107874	0
ENSG00000133636	-1
ENSG00000084774	-1
ENSG00000123892	-1
ENSG00000134982	1
ENSG00000065970	0
ENSG00000125255	0
ENSG00000176749	1
ENSG00000158825	-1
ENSG00000125046	-1
ENSG00000100249	0
ENSG00000129226	0
ENSG00000197632	-1
ENSG00000136997	-1
ENSG00000111667	0
ENSG00000099622	0
ENSG00000143546	1
ENSG00000196345	0
ENSG00000023287	0
ENSG00000134812	0
ENSG00000108861	0
ENSG00000118503	-1

ENSG00000125462	1
ENSG00000081051	-1
ENSG00000146463	0
ENSG00000215251	0
ENSG00000164690	0
ENSG00000197329	0
ENSG00000088992	0
ENSG00000112062	0
ENSG00000117394	0
ENSG00000147588	1
ENSG00000175336	-1
ENSG00000123843	-1
ENSG00000113494	0
ENSG00000106927	-1
ENSG00000130957	1
ENSG00000180447	0
ENSG00000139329	-1
ENSG00000142655	0
ENSG00000152595	1
ENSG00000125637	-1
ENSG00000176928	0
ENSG00000169194	0
ENSG00000140986	-1
ENSG00000129194	1
ENSG00000143106	0
ENSG00000135679	0
ENSG00000013275	0
ENSG00000159423	0
ENSG00000047457	0
ENSG00000127831	-1
ENSG00000108309	1
ENSG00000106153	0
ENSG00000101204	1
ENSG00000129158	0
ENSG00000156299	1
ENSG00000115561	0
ENSG00000126561	-1
ENSG00000083937	0
ENSG00000104435	1
ENSG00000129038	-1
ENSG00000123240	0
ENSG00000161905	-1
ENSG00000131095	1
ENSG00000171811	1
ENSG00000147488	1
ENSG00000127588	1
ENSG00000164111	-1

ENSG00000175866	1
ENSG00000172733	1
ENSG00000138356	-1
ENSG00000175264	1
ENSG00000172209	1
ENSG00000186951	0
ENSG00000185920	0
ENSG00000196104	1
ENSG00000172775	0
ENSG00000082126	0
ENSG00000163798	0
ENSG00000118777	0
ENSG00000115163	-1
ENSG00000197540	-1
ENSG00000118900	0
ENSG00000015592	1
ENSG00000163406	1
ENSG00000165973	1
ENSG00000110321	0
ENSG00000159784	1
ENSG00000257335	0
ENSG00000243147	0
ENSG00000113070	0
ENSG00000096968	0
ENSG00000100095	1
ENSG00000130287	1
ENSG00000171509	1
ENSG00000171956	0
ENSG00000066697	0
ENSG00000092531	0
ENSG00000198171	0
ENSG00000118600	0
ENSG00000152518	0
ENSG00000008438	0
ENSG00000142871	-1
ENSG00000140379	-1
ENSG00000130396	0
ENSG00000110080	-1
ENSG00000173110	0
ENSG00000125457	0
ENSG00000143653	0
ENSG00000162989	1
ENSG00000125648	0
ENSG00000178031	0
ENSG00000077080	1
ENSG00000100321	1
ENSG00000165553	1

ENSG00000137413	0
ENSG00000109606	0
ENSG00000156639	0
ENSG00000188338	0
ENSG00000138381	0
ENSG00000165887	0
ENSG00000158092	0
ENSG00000169676	0
ENSG00000126010	0
ENSG00000157456	-1
ENSG00000120949	-1
ENSG00000146729	0
ENSG00000145649	0
ENSG00000043591	1
ENSG00000066044	0
ENSG00000256294	0
ENSG00000204290	0
ENSG00000115942	0
ENSG00000003402	0
ENSG00000129535	0
ENSG00000105679	0
ENSG00000111348	-1
ENSG00000168078	-1
ENSG00000161558	0
ENSG00000139842	0
ENSG00000143257	0
ENSG00000114383	0
ENSG00000035720	-1
ENSG00000124762	0
ENSG00000167286	-1
ENSG00000149554	-1
ENSG00000088543	1
ENSG00000034510	0
ENSG00000276935	0
ENSG00000152822	1
ENSG00000117595	1
ENSG00000051180	-1
ENSG00000261609	0
ENSG00000006062	0
ENSG00000196381	1
ENSG00000124557	0
ENSG00000164761	-1
ENSG00000138399	0
ENSG00000090889	-1
ENSG00000113905	0
ENSG00000124568	-1
ENSG00000136535	1

ENSG00000140675	0
ENSG00000076604	-1
ENSG00000142910	0
ENSG00000155189	0
ENSG00000133107	1
ENSG00000163814	-1
ENSG00000161649	0
ENSG00000186298	0
ENSG00000101751	0
ENSG00000110330	0
ENSG00000137274	0
ENSG00000130489	0
ENSG00000160870	-1
ENSG00000239672	0
ENSG00000006831	0
ENSG00000114738	0
ENSG00000156983	0
ENSG00000104808	0
ENSG00000204650	0
ENSG00000169006	1
ENSG00000205220	0
ENSG00000172869	0
ENSG00000137491	1
ENSG00000067533	0
ENSG00000108381	1
ENSG00000165023	1
ENSG00000089199	1
ENSG00000164451	-1
ENSG00000128052	1
ENSG00000139618	-1
ENSG00000130052	0
ENSG00000161281	1
ENSG00000109424	0
ENSG00000269404	-1
ENSG00000113889	-1
ENSG00000077238	0
ENSG00000126778	-1
ENSG00000130656	-1
ENSG00000104660	0
ENSG00000173503	0
ENSG00000131482	-1
ENSG00000112195	0
ENSG00000123485	-1
ENSG00000134285	-1
ENSG00000151729	1
ENSG00000066654	0
ENSG00000123384	0

ENSG00000115457	-1
ENSG00000126861	1
ENSG00000183837	1
ENSG00000135452	0
ENSG00000196420	0
ENSG00000162434	0
ENSG00000021461	0
ENSG00000185615	1
ENSG00000130940	0
ENSG00000112984	-1
ENSG00000162928	0
ENSG00000091879	1
ENSG00000142168	0
ENSG00000131187	-1
ENSG00000073578	0
ENSG00000145050	0
ENSG00000181856	0
ENSG00000132840	0
ENSG00000132646	-1
ENSG00000169696	0
ENSG00000005981	0
ENSG00000103035	0
ENSG00000113838	0
ENSG00000115138	0
ENSG00000117362	0
ENSG00000170537	0
ENSG00000120662	0
ENSG00000196387	0
ENSG00000104722	1
ENSG00000189037	-1
ENSG00000110484	0
ENSG00000132964	0
ENSG00000177606	0
ENSG00000145192	-1
ENSG00000134333	-1
ENSG00000104218	0
ENSG00000134716	1
ENSG00000135046	-1
ENSG00000198315	0
ENSG00000136108	0
ENSG00000106952	1
ENSG00000162931	1
ENSG00000106328	0
ENSG00000125746	0
ENSG00000163050	0
ENSG00000175348	0
ENSG00000110583	0

ENSG00000182187	0
ENSG00000105374	0
ENSG00000107833	-1
ENSG00000112081	0
ENSG00000186231	1
ENSG00000186184	0
ENSG00000168938	-1
ENSG00000070770	0
ENSG00000198798	-1
ENSG00000130598	-1
ENSG00000186310	1
ENSG00000100156	1
ENSG00000101986	-1
ENSG00000141433	1
ENSG00000049245	0
ENSG00000129514	-1
ENSG00000169495	0
ENSG00000197056	0
ENSG00000134602	-1
ENSG00000196511	0
ENSG00000167977	0
ENSG00000142149	0
ENSG00000124588	0
ENSG00000105523	0
ENSG00000137673	0
ENSG00000112337	-1
ENSG00000148308	0
ENSG00000120708	-1
ENSG00000157423	1
ENSG00000131437	1
ENSG00000129559	0
ENSG00000196092	-1
ENSG00000166033	0
ENSG00000157796	0
ENSG00000006282	0
ENSG00000147408	0
ENSG00000155897	1
ENSG00000112685	0
ENSG00000090621	-1
ENSG00000162552	0
ENSG00000132535	1
ENSG00000185254	0
ENSG00000178607	-1
ENSG00000066777	0
ENSG00000132002	0
ENSG00000116039	-1
ENSG00000162692	0

ENSG00000197746	-1
ENSG00000083750	0
ENSG00000145703	-1
ENSG00000163993	-1
ENSG00000126767	0
ENSG00000175535	0
ENSG00000133020	0
ENSG00000007402	1
ENSG00000157219	1
ENSG00000136040	0
ENSG00000183625	-1
ENSG00000178568	1
ENSG00000165458	-1
ENSG00000173406	1
ENSG00000159202	0
ENSG00000152049	0
ENSG00000102678	1
ENSG00000135903	0
ENSG00000112182	0
ENSG00000178966	0
ENSG00000135220	-1
ENSG00000162992	1
ENSG00000147571	1
ENSG00000165417	0
ENSG00000198088	-1
ENSG00000148926	-1
ENSG00000136244	0
ENSG00000133321	0
ENSG00000172071	0
ENSG00000072506	-1
ENSG00000106829	0
ENSG00000044012	0
ENSG00000139220	1
ENSG00000125735	-1
ENSG00000089248	0
ENSG00000095539	0
ENSG00000127329	0
ENSG00000119414	0
ENSG00000176390	0
ENSG00000143748	0
ENSG00000071575	0
ENSG00000106100	0
ENSG00000119048	0
ENSG00000184640	0
ENSG00000136444	0
ENSG00000125538	0
ENSG00000138378	1

ENSG00000167657	0
ENSG00000178662	1
ENSG00000166888	0
ENSG00000108960	0
ENSG00000091262	-1
ENSG00000112164	0
ENSG00000163521	0
ENSG00000140564	-1
ENSG00000197208	0
ENSG00000268194	-1
ENSG00000114520	0
ENSG00000143365	0
ENSG00000160460	1
ENSG00000115461	0
ENSG00000108588	0
ENSG00000196544	0
ENSG00000131097	1
ENSG00000240130	-1
ENSG00000204856	0
ENSG00000163541	0
ENSG00000064835	0
ENSG00000181896	0
ENSG00000115604	0
ENSG00000121594	0
ENSG00000126583	1
ENSG00000196323	0
ENSG00000115540	0
ENSG00000130427	-1
ENSG00000126787	-1
ENSG00000138376	-1
ENSG00000076924	0
ENSG00000101928	0
ENSG00000069943	0
ENSG00000179218	0
ENSG00000066135	0
ENSG00000122861	-1
ENSG00000148702	1
ENSG00000058866	1
ENSG00000177951	0
ENSG00000151806	0
ENSG00000047597	0
ENSG00000107968	0
ENSG00000132109	0
ENSG00000165392	0
ENSG00000135740	0
ENSG00000180532	0
ENSG00000147166	-1

ENSG00000144834	1
ENSG00000146378	-1
ENSG00000108878	0
ENSG00000163221	1
ENSG00000196839	-1
ENSG00000145569	0
ENSG00000066056	0
ENSG00000169814	0
ENSG00000149489	1
ENSG00000160049	0
ENSG00000059915	1
ENSG00000139626	-1
ENSG00000023228	0
ENSG00000100197	0
ENSG00000112077	-1
ENSG00000087245	-1
ENSG00000091651	-1
ENSG00000163815	1
ENSG00000099849	0
ENSG00000070388	0
ENSG00000108352	1
ENSG00000134262	0
ENSG00000100528	0
ENSG00000169413	-1
ENSG00000078900	0
ENSG00000122971	0
ENSG00000128965	0
ENSG00000111732	0
ENSG00000138814	1
ENSG00000175224	0
ENSG00000134853	0
ENSG00000120215	-1
ENSG00000151655	-1
ENSG00000169738	0
ENSG00000125084	0
ENSG00000138109	0
ENSG00000131759	0
ENSG00000110696	0
ENSG00000167244	-1
ENSG00000153002	0
ENSG00000121931	-1
ENSG00000076554	0
ENSG00000172238	-1
ENSG00000110011	0
ENSG00000149311	0
ENSG00000076662	-1
ENSG00000139372	0

ENSG00000055955	0
ENSG00000078401	0
ENSG00000056558	0
ENSG00000100908	0
ENSG00000136982	-1
ENSG00000131781	-1
ENSG00000146678	-1
ENSG00000160789	-1
ENSG00000196352	-1
ENSG00000132693	0
ENSG00000073921	0
ENSG00000135945	0
ENSG00000128050	-1
ENSG00000160182	-1
ENSG00000183486	-1
ENSG00000163161	0
ENSG00000125257	-1
ENSG00000147100	0
ENSG00000154620	1
ENSG00000141946	0
ENSG00000152583	1
ENSG00000139679	0
ENSG00000135423	1
ENSG00000139687	0
ENSG00000116478	-1
ENSG00000131398	0
ENSG00000168216	1
ENSG00000151090	1
ENSG00000171033	1
ENSG00000120685	0
ENSG00000166803	-1
ENSG00000156265	0
ENSG00000094755	-1
ENSG00000007908	1
ENSG00000137745	-1
ENSG00000115648	-1
ENSG00000135547	0
ENSG00000136156	0
ENSG00000023839	-1
ENSG00000026652	1
ENSG00000152229	-1
ENSG00000085563	1
ENSG00000105143	1
ENSG00000141527	-1
ENSG00000167720	0
ENSG00000039068	-1
ENSG00000123576	-1

ENSG00000110060	0
ENSG00000155957	0
ENSG00000104907	0
ENSG00000145863	1
ENSG00000123505	0
ENSG00000196368	0
ENSG00000124641	0
ENSG00000197081	0
ENSG00000144827	0
ENSG00000006210	0
ENSG00000141293	-1
ENSG00000187730	1
ENSG00000141526	-1
ENSG00000072274	-1
ENSG00000160145	1
ENSG00000110921	0
ENSG00000174015	0
ENSG00000066923	-1
ENSG00000079102	1
ENSG00000106330	0
ENSG00000148154	0
ENSG00000151116	0
ENSG00000096006	-1
ENSG00000197446	0
ENSG00000104067	0
ENSG00000129562	0
ENSG00000072778	0
ENSG00000075043	0
ENSG00000180245	0
ENSG00000103310	1
ENSG00000022840	0
ENSG00000176890	-1
ENSG00000197635	0
ENSG00000144283	0
ENSG00000135451	-1
ENSG00000164754	0
ENSG00000153395	0
ENSG00000006016	-1
ENSG00000124882	-1
ENSG00000175445	1
ENSG00000197535	1
ENSG00000107262	0
ENSG00000162520	-1
ENSG00000110799	1
ENSG00000117528	0
ENSG00000143476	-1
ENSG00000170323	1

ENSG00000122592	-1
ENSG00000007968	-1
ENSG00000164961	0
ENSG00000087510	-1
ENSG00000130844	0
ENSG00000136045	0
ENSG00000177294	0
ENSG00000144909	0
ENSG00000101115	-1
ENSG00000158164	-1
ENSG00000152785	1
ENSG00000120068	-1
ENSG00000096063	0
ENSG00000114354	0
ENSG00000168036	0
ENSG00000096060	0
ENSG00000150991	0
ENSG00000160007	0
ENSG00000148082	1
ENSG00000124920	1
ENSG00000221978	0
ENSG00000073756	0
ENSG00000145020	0
ENSG00000125522	0
ENSG00000114013	0
ENSG00000091583	-1
ENSG00000161542	0
ENSG00000244165	0
ENSG00000070501	0
ENSG00000136694	0
ENSG00000152503	1
ENSG00000176076	1
ENSG00000163867	0
ENSG00000120251	1
ENSG00000065135	0
ENSG00000000938	1
ENSG00000113594	-1
ENSG00000135119	0
ENSG00000119487	0
ENSG00000145740	0
ENSG00000124194	1
ENSG00000164885	0
ENSG00000132773	0
ENSG00000132170	0
ENSG00000154975	1
ENSG00000163513	0
ENSG00000163820	0

ENSG00000075239	0
ENSG00000140598	0
ENSG00000197838	0
ENSG00000163703	0
ENSG00000070915	-1
ENSG00000119139	0
ENSG00000152464	0
ENSG00000091704	0
ENSG00000139629	0
ENSG00000148444	0
ENSG00000071246	1
ENSG00000118513	-1
ENSG00000115594	0
ENSG00000110917	0
ENSG00000151702	0
ENSG00000164626	-1
ENSG00000104537	0
ENSG00000108852	1
ENSG00000167196	0
ENSG00000128590	0
ENSG00000075618	0
ENSG00000182631	0
ENSG00000169918	1
ENSG00000036530	1
ENSG00000124575	-1
ENSG00000140465	-1
ENSG00000138180	-1
ENSG00000009724	0
ENSG00000140987	0
ENSG00000179477	-1
ENSG00000128886	0
ENSG00000184674	0
ENSG00000196811	0
ENSG00000130544	0
ENSG00000100644	0
ENSG00000169047	0
ENSG00000186205	0
ENSG00000102539	0
ENSG00000163235	0
ENSG00000188785	0
ENSG00000108231	1
ENSG00000128563	0
ENSG00000105388	-1
ENSG00000084674	-1
ENSG00000172534	0
ENSG00000078142	0
ENSG00000105053	0

ENSG00000197442	1
ENSG00000169371	0
ENSG00000177463	0
ENSG00000048028	0
ENSG00000075213	0
ENSG00000067221	1
ENSG00000135446	-1
ENSG00000185624	-1
ENSG00000120211	-1
ENSG00000138185	1
ENSG00000084207	0
ENSG00000161634	0
ENSG00000180209	0
ENSG00000071539	-1
ENSG00000151079	1
ENSG00000116815	-1
