# Supplementary material for: RNA-Seq differential expression analysis: An extended review and a software tool
Source: PLoS One. 2017 Dec 21;12(12):e0190152. doi: 10.1371/journal.pone.0190152 (PMC5739479; doi:10.1371/journal.pone.0190152)
Supplement: S2 Table — Performance of each DEGs identification method with different mappers. (PDF) [file pone.0190152.s002.pdf]

Details about the performance[1] of each DEGs identification method with different mappers.

Table 1: NOISeq performance

**NOISeq**					
	**TPR**	**SPC**	**PPV**	**ACC**	F_1 **measure**
**BWA**	0,80	0,94	0,91	0,88	0,85
**Tophat**	0,80	0,95	0,92	0,89	0,86
**Bowtie**	0,80	0,95	0,92	0,89	0,86

Table 2: DESeq performance

**DESeq**					
	**TPR**	**SPC**	**PPV**	**ACC**	F_1 **measure**
**BWA**	0,44	0,58	0,42	0,52	0,43
**Tophat**	0,44	0,59	0,43	0,53	0,44
**Bowtie**	0,44	0,59	0,43	0,53	0,44

Table 3: baySeq performance

**baySeq**					
	**TPR**	**SPC**	**PPV**	**ACC**	F_1 **measure**
**BWA**	0,92	0,40	0,52	0,61	0,66
**Tophat**	0,92	0,40	0,52	0,61	0,66
**Bowtie**	0,92	0,39	0,52	0,61	0,66

Table 4: edgeR performance

**edgeR**					
	**TPR**	**SPC**	**PPV**	**ACC**	F_1 **measure**
**BWA**	0,72	0,94	0,89	0,85	0,79
**Tophat**	0,71	0,94	0,90	0,85	0,79
**Bowtie**	0,71	0,94	0,90	0,85	0,79

[1] TPR: True Positive Value. SPC: Specificity. PPV: Positive Predict Value. ACC: Accuracy.

Table 5: edgeR performance with other methods

**edgeR**					
**Methods**	**TPR**	**SPC**	**PPV**	**ACC**	F_1 **measure**
Bowtie2	0,71	0,94	0,90	0,85	0,79
BWA	0,72	0,94	0,89	0,85	0,79
TopHat	0,71	0,94	0,90	0,85	0,79
Salmon	0,71	0,93	0,88	0,84	0,78
STAR	0,82	0,95	0,91	0,89	0,86
kallisto	0,74	0,91	0,86	0,84	0,79

Table 6: NOISeq performance with other methods

**NOISeq**					
**Methods**	**TPR**	**SPC**	**PPV**	**ACC**	F_1 **measure**
Bowtie2	0,67	0,94	0,89	0,83	0,76
BWA	0,66	0,94	0,89	0,83	0,76
TopHat	0,80	0,95	0,92	0,89	0,86
Salmon	0,67	0,90	0,83	0,81	0,74
STAR	0,68	0,96	0,93	0,85	0,78
kallisto	0,67	0,92	0,85	0,81	0,75
